# Does capping social security harm health? A natural experiment in the UK

**DOI:** 10.1111/spol.12768

**Published:** 2021-09-08

**Authors:** Aaron Reeves, Mark Fransham, Kitty Stewart, Ruth Patrick

**Affiliations:** ^1^ Department of Social Policy and Intervention University of Oxford Oxford UK; ^2^ International Inequalities Institute, LSE London UK; ^3^ Centre for Analysis of Social Exclusion, LSE London UK; ^4^ Department of Social Policy University of York York UK

**Keywords:** benefit cap, mental health, social security, welfare reform

## Abstract

In this paper, we examine the mental health effects of lowering the UK's benefit cap in 2016. This policy limits the total amount a household with no‐one in full‐time employment can receive in social security. We treat the reduction in the cap as a natural policy experiment, comparing those at risk of being capped and those who were not, and examining the risk of experiencing poor mental health both before and after the cap was lowered. Drawing on data from ~900,000 individuals, we find that the prevalence of depression or anxiety among those at risk of being capped increased by 2.6 percentage points (95% confidence interval: 1.33–3.88) compared with those at a low risk of being capped. Capping social security may increase the risk of mental ill health and could have the unintended consequence of pushing out‐of‐work people even further away from the labour market.

## 1 INTRODUCTION

The logic of social security systems in some high‐income countries has shifted in recent decades, leading to a reconfiguration of the conditions of entitlements and the structure of the financial support offered to those in precarious economic circumstances (Hills, 2014; Pierson, 1994). Social security in the UK was originally organised around the principle of ‘contribution in return for benefits’ to provide a ‘minimum’ income ‘as of right… so that individuals may build freely upon it’ (Timmins, 2017, pp. 23–24). These more recent reforms have been motivated by a desire to reduce the cost of this system but more recent reformers are also far more concerned than the architects of the welfare state about the possibility that overly generous welfare regimes foster a culture of dependency among recipients (Hills, 2014). One frequent manifestation of this dependence logic has been policy interventions that make social security less generous in order to activate labour market participation. Critics of this approach, however, have raised concerns that these reforms may have unintended consequences, such as harming mental health, increasing poverty and exacerbating stress (Morris, 2008).

In this paper, we focus on the potential unintended consequences of one policy that is rooted in this logic of welfare reform—the benefit cap. Beyond a direct concern about the well‐being of the individuals affected, the mental health effects of the benefit cap matter for four main reasons. First, we would traditionally expect social security interventions to aspire to improving the mental health of affected populations; and so it is important to assess the impact the cap has on adult mental health. Second, if the mental health of parents declines then this may have negative effects for their children too, potentially adversely affecting their well‐being, educational development and behaviour (Cooper & Stewart, 2020). Third, worsening mental health may actually push people away from the labour market, in that mental health problems can decrease the likelihood of returning to work (García‐Gómez et al., 2010). The cap may therefore have the unintended effect of deepening the degree of social exclusion felt by some of these individuals. Fourth, worsening mental health incurs costs for government. This is particularly salient in the UK where healthcare is almost entirely tax‐financed. Further, while the benefit cap is somewhat unusual, it has some similarities to other policies which establish limits on welfare spending, such as block‐grants (Ziliak, 2015) or time limits (e.g., the 60‐month federal limit for Temporary Assistance for Needy Families; Grogger, 2002). In this respect, analysing the mental health effects of the benefit cap may have implications for welfare limits more generally.

To date, however, there have been no evaluations of the mental health consequences of the benefit cap. On the one hand, negative effects may be expected, as the policy is intended to reduce family income and we know that income is correlated with mental health (Cooper & Stewart, 2015). While there are still relatively few studies that use causal or quasi‐experimental techniques to investigate these links (Simpson et al., 2021), the literature points to a causal relationship between low income and mental health, and especially between low income and maternal mental health (Cooper & Stewart, 2015; Cooper & Stewart, 2020). This last point is pertinent as a high percentage of capped households are larger families headed by a lone mother. However, many of the existing studies in this area look at increases rather than cuts to income, while those that have examined the mental health effects of reductions in social security have not focused on lone parents (Reeves et al., 2016). Sudden reductions in benefit income are unusual and therefore little investigated, and we cannot assume they will simply have the reverse effect of an increase in income.

At the same time, the benefit cap is potentially rather different to other cuts to social security benefits, as the cap was deliberately set initially at roughly ‘the average take home pay of working households’ (DWP, 2014, p. 11). The explicit justification, set out by the Chancellor of the Exchequer, George Osborne, in announcing the policy in October 2010, was to ensure that ‘no family should get more from living on benefits than the average family gets from going out to work’ (Osborne, 2010). If the cap is set close to median earnings, one might expect even the capped amount to provide a reasonable minimum income for those families affected, and therefore the policy might be unlikely to harm mental health in the same way as a cut to benefits for those already living in hardship; indeed, in providing an incentive to move into work it could plausibly even lead to mental health improvements.

Critics, however, have noted that equating an out‐of‐work family's *total income* with an in‐work family's *earnings* ignores differences between families in their composition and therefore spending needs. Critically, it also overlooks the additional support many in‐work families are entitled to, which increase their *total income*, and help them meet their needs, for example, through child benefits and housing support. Thus, depending on whether any capped families have been enjoying levels of income that exceed family needs, the cap may have relatively small (perhaps even positive) effects on mental health for affected individuals, or it may be damaging.

We address this gap in our understanding by treating a change in the level of the benefit cap in late 2016 as a natural experiment (Dunning, 2012). We draw on a large, repeated cross‐sectional sample survey to identify those who are at risk of being subject to the cap and those who are not. We then follow these groups over time and using a variety of causal identification strategies (including difference‐in‐differences models and interrupted time series analysis) we show that lowering the level of the cap (and thereby increasing the number of people who were at risk of being affected, as well as the size of the income loss for those already affected) increased the risk of reporting mental ill health. Our results suggest that the mental health effects of income shocks, such as reductions in social security, need to be viewed alongside the possible labour market effects. While those affected by the benefit cap may be more likely to move into work, our evidence also suggests that this policy may actually push people further away from the labour market because it undermines their well‐being.

### 1.1 The benefit cap and welfare reform in the UK

The Conservative‐led Coalition Government was elected in 2010 on a commitment to reduce government spending and, soon after taking office, they announced a raft of reforms aimed at reducing social security expenditures (Osborne, 2010). The benefit cap was part of this policy programme and was initially set at £500 per week (or £26,000 per year) for couples and lone parents, and an equivalent amount of £350 per week (or £18,200 per year) for single people without children (or whose children do not live with them). The rate for couples was set on the basis of estimated median earnings for working households after tax and national insurance, with the single rate set at 70% of the couple rate, broadly in line with OECD equivalence scales (DWP, 2014). The cap applied only to ‘workless families’, with exemptions also for families in receipt of disability benefits. In practice, the households most likely to be affected were lone parents and larger families who were living in private renter accommodation.

Implementation started in April 2013 but accelerated from July 2013 onwards. Most of those going onto the cap did not know much about it (despite having heard of the policy) and there seems to have been some confusion around whether people would be affected or not (Finlay et al., 2013). By the end of that year, however, around 28,000 families were subject to the cap every month. The government's 1 year review of the cap was glowing, noting that ‘we did not fully appreciate the scale of the positive benefits of the cap’ (DWP, 2014). Preliminary evidence suggested the policy successfully motivated capped individuals to look for work or, if they had already been seeking work, to look harder (Kaur et al., 2014). The review argued that these changes would deliver wider benefits for society, such as discouraging ‘benefit dependency’ and ‘break[ing] intergenerational cycles of disadvantage’ (DWP, 2014). The policy was popular too, with almost 75% of people in some polling data suggesting that people were in favour of the policy (Finlay et al., 2013).

A few years later, in 2015, the Conservative Party included a commitment to reduce the cap further in their general election manifesto; this reduction was confirmed in the Summer Budget 2015, after the Conservatives had been returned to power with a majority. In November 2016, the cap was reduced from £26,000 per year to £23,000 per year for families in London (£15,410 for single people) and to £20,000 (13,400 for single people) outside the capital.^1^ By March 2017, around 68,000 families were subject to the cap each month. In total, more than 290,000 families had their benefit payments capped between April 2013 and November 2019. The cap targeted spending reductions and work incentives on a group whose entitlements were perceived to be prima facie too large and who were, in principle, able to work.

There are a number of exemptions from the cap. Pensioners, those claiming working tax credits (WTC), and families with at least one adult receiving financial support because of a disability that stops them from working are all exempt. There are also informal exemptions too. For example, a claimant's local authority may temporarily offer them Discretionary Housing Payments, which provide transitionary support for those struggling to find work or to move to a more affordable property.

The cap has always been controversial because it disproportionately affects particular groups. Initially, for example, many capped families (over 40%) were based in London, where rents are high. Since the cap became more restrictive, its effects have reached almost every part of the country (only one quarter of capped families in 2017 were in London). The cap also disproportionately affects women and children. More than 70% of capped families are single parents and most of these are headed by women (~90%) (DWP, 2020). In total, over 93% of capped families include children and the majority are larger families with three or more children (DWP, 2020).

**FIGURE 1:**
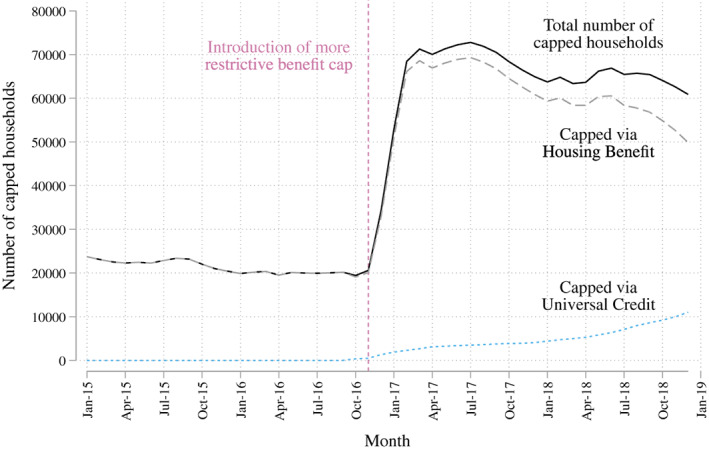
Number of households subject to the benefit cap between 2015 and 2018. *Source*: Office for National Statistics. People can be capped in two different ways, both represented on the graph. Most people are capped through their Housing Benefit (the financial support they receive for their housing) but others are capped through Universal Credit, a new benefit system which combines out‐of‐work support, tax credits, and housing benefit [Colour figure can be viewed at wileyonlinelibrary.com]

The economic shock experienced by capped families is not trivial. Once households have been judged to be subject to the cap (see Figure 1) their income from Housing Benefit or Universal Credit will fall in the months following. On average, those affected by the benefit cap lose around £2600 per year, slightly more for families with children (DWP, 2020). This is approximately a 10% reduction in total family income, and would equate to a higher share of disposable income after housing costs, if part of the benefit package is covering rent and the family does not move (more details on who is affected and where they live can be found in Appendix S1). This potentially places families under increasing pressure in seeking to make ends meet. Very few people subject to the cap move to a new property and so the remaining options are either to look for a job (often while managing childcare) or to reduce spending (DWP, 2020). Early indications suggest that, between 2018/2019 and 2023/2024, the benefit cap will push around 400,000 children deeper into poverty (Tucker, 2019), and these estimates do not take into account the sharp increases in the numbers of capped families as a result of the effects of Covid‐19.

### 1.2 How the benefit cap could affect mental health

What are the mechanisms that potentially link a reduction in social security receipt and mental health? One possibility is that the cap has no negative effect on mental health and even some positive effects. If, as ministers implied when the policy was announced, those now capped were actually not facing economic difficulties, then we might anticipate limited substantial impact on their well‐being (Osborne, 2010), especially if the impact of income on well‐being diminishes at higher levels of income (Cooper & Stewart, 2015). Moreover, the policy was intended to incentivise people back into work and, because work can be good for health, some people may actually experience improvements in their well‐being (Bartley, 1994).

In contrast, there are several reasons why we might expect harm to mental health as a result of the cap. First, despite the rhetoric, the benefit cap affects households that are living below or just above the poverty line. This reduction in income may limit families' ability to buy essential goods and services which could directly affect mental as well as physical well‐being. Second, beyond basic necessities, a difficulty in covering wider household expenses, including ensuring that children have what they need for school and for social participation, can worsen mental health through increased stress: there is strong evidence of a causal relationship between poverty and maternal depression (Cooper & Stewart, 2015; Cooper & Stewart, 2020). Third, economic stress may lead to conflict and irritability in relations in the household, both between adults and between adults and older children (Conger et al., 1994) and this in turn may affect well‐being. A fourth mechanism is related to longer‐term uncertainty or precariousness (Benach & Muntaner, 2007). Even if, in a given month, a household does manage to cover their costs, the worry or anxiety about managing may be harmful, particularly if sustained. In the case of the benefit cap, there is a serious threat of losing one's home and having to move away from community, schools and support networks, meaning that capped households may be living day‐to‐day with an existential threat to their way of life, which is likely to take its toll on mental health (Clair et al., 2019).

We might also expect the effects from the benefit cap to be cumulative over time: whilst the reduced benefit cap had an immediate impact on family incomes, families may have coped initially with the fall in incomes by drawing on existing resources, including savings or family support, or by seeking temporary Discretionary Housing Payments through the local authority. As these resources diminish over time, we may expect to see the impact of being exposed to the cap increase.

### 1.3 Benefit cap as a natural experiment

In this paper, we use the introduction of the more restrictive benefit cap in November 2016 to examine the causal effect of reducing welfare payments on mental health. We treat this policy change as a natural experiment, exploiting the fact that those exposed to the cap experienced a sudden drop in income which did not affect other households. We focus on the point at which the cap was lowered, rather than the cap's initial introduction in 2013, because a much larger number of households were affected by the 2016 reform, while the size of the average impact on capped families increased.

We note that any impact on mental health may not show up in the data immediately but may instead emerge gradually and that there are theoretical reasons why the policy may *not* have harmful effects. This specific policy change, then, allows us to test whether the families most likely to be affected by the reform—lone parents with children—can lose this extra income without harm. To date, we do not have good evidence on whether or not the benefit cap will harm mental health, and this paper attempts to fill this gap.

## 2 DATA AND METHOD

We use two large‐scale, repeated cross‐sectional surveys from the UK, both of which are stratified, random samples of private addresses used to produce official statistics. Response rates are typically over 50%. The first (and primary) data set is the UK Labour Force Survey (LFS), which interviews 90,000 people quarterly. The second data set is the Family Resources Survey (FRS). This is the highest quality survey capturing household income from all sources in the UK and contains around 20,000 families annually. As the FRS does not contain measures of mental health, we focus primarily on the LFS, using the FRS for sensitivity analysis, as explained below.

The LFS does not contain a way of formally identifying those who have been capped or not and so we adopt an intention‐to‐treat approach: we identify those who are *at risk* of being capped and compare them with those who are not. We define families as at risk of being capped if they are: aged 16–65, in rented accommodation, either a lone parent (with any number of children) or a two‐parent family which contains at least three dependent children, and receiving housing benefit and at least two other form of social security (e.g., Income Support or Jobseeker's Allowance). We exclude those who meet all of these criteria but are in receipt of WTC, as households with a member in receipt of WTC are exempt. There are a very small number of people who are in work but who are not receiving WTC (*n* = 30), presumably because they have not claimed it. We focus on lone parents and large families in rented accommodation because these are the main risk factors for being capped (DWP, 2020). Everyone else is initially included in the comparison group. This is an imperfect contrast, of course, and so we also explore whether our results change when we focus on particular subgroups of this broader comparison group.

Our main measure suggests around 0.76% of our sample were at risk of being capped (*n* = 6824). This is ~3 times larger than the proportion of capped households in the overall population (~0.25%) and potentially introduces bias into our results because there will be measurement error in the ‘treatment’ variable. However, if anything, this intention‐to‐treat approach is likely to be more conservative because it means there will be uncapped households who are treated as capped in our analysis, and this is likely to push our results towards zero. Despite this measurement error, those categorised as at risk of being capped in our data are, as expected, predominantly women (~95%) in their mid‐30s, who are lone parents (~97%) and economically inactive (~73%). In fact, the sample we identify as being at risk of being capped looks similar to the administrative data on capped families in terms of age, gender, whether a child is present in the home, and whether or not it is a single adult family (Appendix S2). There are some differences, however. Our sample has a lower share of people in London, a higher share of lone parents, and the average number of children is smaller.

Importantly, our measure of at risk individuals is largely stable before and after the reforms was implemented (see Appendix S2 for balance tests). There was a decline in the proportion of those at risk living in London and there was a slight decline in the proportion of people who were unemployed and looking for work. It is important to note that the composition of capped households in the administrative data was also broadly stable before and after the cap was lowered, with the exception of the expected decrease in the share of capped households who live in London (see Appendix S2). No data on economic status is available in the administrative data.

Our measure of mental health is drawn from a battery of health questions in which respondents are asked to affirm whether they have (no time period is specified) ‘depression, bad nerves or anxiety’. In effect, our measure of mental health is a binary variable with 1 representing those who describe themselves as having depression and anxiety and 0 otherwise, and is not necessarily connected to a formal diagnosis. Respondents are also asked about 16 other health conditions, including breathing difficulties, difficulties hearing or seeing, arthritis, high blood pressure, epilepsy, and diabetes. There is no limit to the number of illnesses they can select from this list. We create a variable for poor physical health which codes as 1 those individuals who report experiencing any of these other non‐depression/anxiety‐related illnesses. The physical health variable is used in a falsification test, described below.

### 2.1 Statistical analysis

In the few months following the lowering of the benefit cap in November 2016, the numbers being capped increased rapidly (see Figure 1). This meant that many more families were exposed to the cap, while families that were already capped experienced additional reductions in their incomes. We now examine the mental health of those at risk of being capped before and after November 2016, and compare those at‐risk of being capped with those who experienced a low risk according to our indicator.

We estimate an OLS difference‐in‐differences model with the following specification:

$${\text{P(Health}}_{i,t})=\alpha +\beta_{1}{\text{Capped}}_{i}+\beta_{2}{\text{Policy}}_{t}+\beta_{3}{\text{Capped}}_{i}\times {\text{Policy}}_{t}+\beta_{z}X_{i}+\varepsilon_{i,t}$$

where *i* denotes individuals and *t* the time‐period in which the data were collected. *Health* is a binary measure which is 1 if respondents report experiencing ‘depression and anxiety’ from a list of possible health problems and 0 otherwise. *α* is the constant (which in the model reports the probability of experiencing depression‐like symptoms before the policy change and among those at a low risk of being capped). *Capped* is a dummy variable which is 1 if the respondent meets the criteria described above for being at risk of being capped (*n* = 6824) and 0 otherwise (*n* = 893,682). *Policy* is 1 if an individual was interviewed during or after November 2016 (*n* = 460,364) and 0 otherwise (*n* = 440,142). Capped × Policy is an interaction term which captures those who are at risk of being capped and who are interviewed after the cap has become more restrictive. *X*_*i*_ is a vector of control variables. These include age (measured in years), gender, the government office region in which the respondent lives, whether they self‐report being ‘white’ in a question about ethnicity, whether they have other health problems aside from ‘depression and anxiety’, whether they are a renter or not, education (seven categories ranging from university degree to no qualifications), and their economic status (whether they were employed, unemployed, or economically inactive). *ε* is our error term. Our standard errors are clustered at the level of the treatment period, a conservative approach (Brewer et al., 2018).

Our coefficient of interest is *β*_3_—the difference‐in‐differences estimate. If *β*_3_ > 0 then those at risk of being capped faced a higher probability of experiencing ‘depression and anxiety’ after the reform over and above any change in those with a lower risk of being capped.

Our identification strategy is to assume that, in the absence of the benefit cap reform, the trends in reporting anxiety or depression among the ‘at risk of being capped’ group would have been identical to the trend for the ‘not at risk’ group. We test the parallel trends assumption using a variety of approaches, including testing whether results are consistent when adjusting for the predicted linear trend and changing associations between covariates and the intervention (see Appendix S3). We also re‐estimate our main models using an interrupted time series design to test whether the trends in mental health diverge after November 2016, when the cap becomes more restrictive. This confirms the parallel trends assumption as well as reinforcing our main results.

### 2.2 Sensitivity analyses

We conduct a falsification test to check whether our results are spuriously correlated with processes that should be unrelated to changes in the benefit cap. Here we use the measure of other health conditions described above as our dependent variable. Our theory is that in the short run, while these health conditions could possibly change in response to some policy interventions, they should be uncorrelated with the implementation of a more restrictive benefit cap.

We also explore whether the changes to the benefit cap vary geographically by estimating the same difference‐in‐differences model on the affluent (East Midlands, Eastern, London, South East, Scotland and South West) and then the less affluent parts of the country (North East, North West, Northern Ireland, Yorkshire and Humberside, West Midlands, and Wales), as defined by their gross disposable household income.

We know that capped families are going to be dissimilar to non‐capped families in ways that may affect their mental health and so we estimate a series of models which restrict the households in the ‘comparison’ group to those that are more similar to the capped group than the unrestricted comparison group used in the main analysis. We focus on three contrasts, comparing families at risk of being capped with three other groups who are not subject to the cap: (1) those who are not receiving any benefits but who are renters and have the same household structure (i.e., larger families in rented accommodation but not in receipt of benefits); (2) those who receive benefits but own their home or who are purchasing it through a mortgage, meaning housing costs do not push them into the cap (i.e., larger families who are home owners); and (3) those who are receiving disability benefits and therefore exempt. We also explore a number of other restrictions to our data to examine whether the association is stable across various sub‐groups. We re‐estimate our main models among (1) only lone parents, (2) only households with more than two children, and (3) lone parents with more than two children. We also explore whether excluding those households potentially affected by the two‐child limit alters our results.

One weakness of our main approach is that the LFS does not allow us to clearly identify those who are capped because the LFS does not contain a measure of total income from the government. The Family Resources Survey (FRS), by contrast, has more detailed measures of benefit receipt and therefore allows us to more accurately (albeit still imperfectly) assess who is being capped and who is not. We cannot use this dataset as our main source as it does not include measures of mental health, but as a final sensitivity analysis, we bring together the health data from the LFS with the more accurate benefit cap data from the FRS. To do this, we create a statistical model in the FRS data which predicts whether individuals are likely to be capped or not (see Appendix S4 for full details). We use this model to predict the probability of LFS respondents being capped and then re‐estimate our models using this alternative measure of being at risk of being capped. This enables us to see whether our results remain consistent across these alternative specifications.

## 3 RESULTS

### 3.1 Did the benefit cap harm mental health among those at risk of being capped?

**FIGURE 2:**
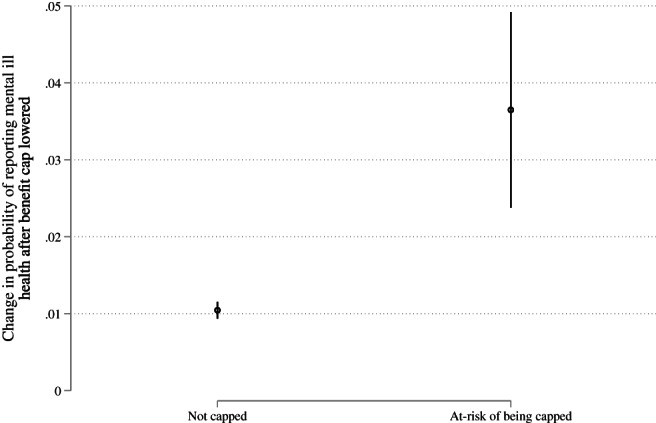
Probability of reporting mental ill health increased more among those at risk of being capped than everyone else after the benefit cap was introduced. Figure based on results from Column 1 in Table 1. Data come from the Labour Force Survey. Vertical lines represent 95% confidence intervals

We start by analysing the LFS data, comparing the probability of reporting depression‐like symptoms among those at risk of being capped and those with a low risk, before and after the reform. Our most basic and unadjusted difference‐in‐differences model suggests that the prevalence of mental health problems increased among those who were at risk of being capped after the reform was introduced and that this increase is greater than what we observe among the rest of the population. Difference‐in‐difference estimates are presented in Table 1 and visualised in Figure 2. Mental ill health is more common at baseline among those who were at risk of being capped: the baseline estimate is ~14 percentage points higher for capped than non‐capped individuals. After the reform, there is a slight increase—1 percentage point—in the prevalence of mental ill health in those not at risk of being capped. But our estimates suggest that among those who were at risk of being capped, the prevalence of mental ill health increased by around 2.6 percentage points over and above this increase in those not at risk of being capped (Model 1: Table 1). Adjusting for our covariates does not alter the main finding (see Model 2: Table 1) nor does excluding individuals that have experienced sustained mental health problems in the past. In other words, we are seeing an increase in the number of people experiencing depression for the first time.

**TABLE 1:** The introduction of the benefit cap increased the prevalence of mental ill health

	Probability of reporting mental health problems		
	(1)	(2)	(3)
Difference‐in‐differences: Capped individuals compared to uncapped individuals after the reform	0.026* (0.0065)	0.023* (0.0061)	0.024* (0.0061)
Change over time for the non‐capped individuals	0.010* (0.00056)	0.011* (0.00052)	0.011* (0.00052)
Difference between capped and non‐capped individuals at baseline	0.14* (0.0042)	0.031* (0.0040)	0.031* (0.0040)
Constant (probability of depression among non‐capped individuals before cap lowered)	0.069* (0.00039)	−0.090* (0.0046)	−0.089* (0.0046)
Controls for covariates		Y	Y
Restrict to those who have never had mental health problem			Y
Number of individuals	900,506	900,481	898,294

*Note*: Standard errors are reported in parentheses. Data come from the Labour Force Survey.

* *p* < 0.01.

**FIGURE 3:**
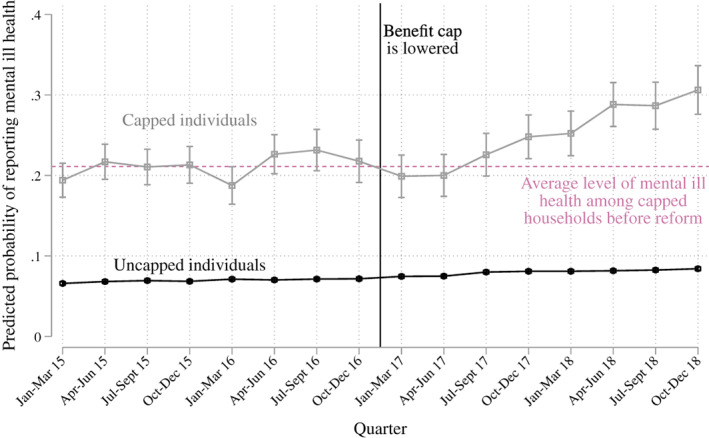
Introduction of the benefit cap and the prevalence of mental ill health among those who are at risk of being capped and those who are not, by quarter. Data come from the Labour Force Survey. Vertical lines represent 95% confidence intervals. Vertical black line indicates when the benefit cap was lowered [Colour figure can be viewed at wileyonlinelibrary.com]

Next, we unpack these difference‐in‐differences estimates by estimating these models not only as a simple before‐and‐after but by examining when precisely the differences emerge. We estimate the prevalence of mental ill health in each quarter of the data, before and after the reform. These estimates are less precise because we only observe ~300 people at risk of being capped in each quarter. Figure 3 shows the results. We find that the level of mental ill health before the reform among those potentially affected by the cap was relatively stable, but after the reform was introduced we see a steady rise in the proportion of people reporting mental ill health. While (as Table 1 suggests) there has been a small increase in the number of people reporting mental ill health among those with a low‐risk of being capped, this has been far less pronounced.

We next consider whether there is regional variation in the impact of the benefit cap (Appendix S5). The cap might be expected to have a larger impact on mental health in areas of the country where housing costs are higher, because the average reduction in income is likely to be higher. On the other hand, the reduction in incomes may be more keenly felt in poorer parts of the country, because the relative reduction might be larger. Even in London, over half (~56%) of capped families lose less than £50 per week, a similar share to the North West (59%), suggesting that the cap might have a larger relative impact in less affluent parts of the country. In fact, our results suggest that the mental health effects of the cuts may have been slightly larger in wealthier parts of the country, where the number of people affected is greater, but this difference is not statistically significant at conventional levels (*p* = 0.15).

**FIGURE 4:**
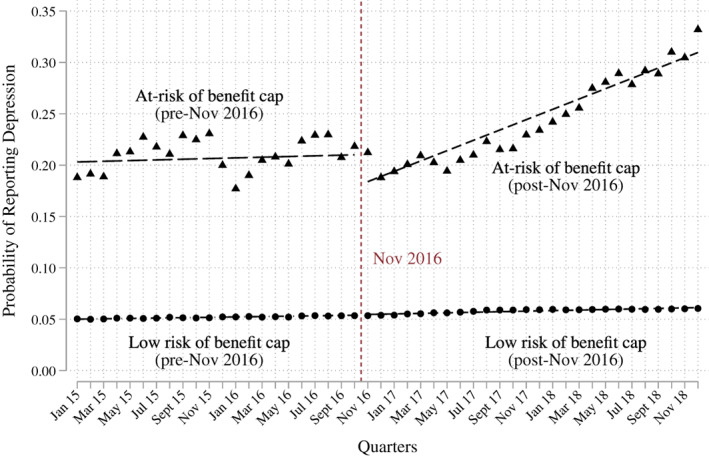
Interrupted time series analysis of the lowering of the benefit cap. Data come from the Labour Force Survey. Each dot represents the 3‐month moving average of the probability of reporting depression. The lines of best fit are extrapolated from the Interrupted Time Series Analysis reported in Appendix S6 [Colour figure can be viewed at wileyonlinelibrary.com]

Finally, we re‐estimate our main finding using an interrupted time series analysis. Here, we calculate the prevalence of mental ill health for both those at risk of being capped and everyone else for every month from January 2015 to December 2018. We then calculate a 3‐month rolling average from these monthly observations. This analysis allows us both to test the parallel trends assumption and to examine whether there is a change in the slopes post reform. The time series models confirm the main findings from our quarterly analysis (Figure 4). We find no difference in the slopes prior to the reform but there is a very clear break in the slope after the reform was introduced, suggesting the trajectory for mental ill health clearly deviates from the rest of the population after the benefit cap becomes more restrictive (Appendix S6 for full results).

### 3.2 Sensitivity tests

Our first sensitivity test is a falsification test. We would not expect the benefit cap to affect other non‐mental health related outcomes over this time‐period and so test whether we find a similar increase in other health problems in our difference‐in‐differences models. Appendix S7 shows that we find no association between those at risk of being capped and other non‐mental health outcomes after the cap was lowered, suggesting our findings are not driven by compositional shifts unaccounted for by our variables nor by some other spurious trend.

Second, we exploit various exclusions to the benefit cap to see whether restricting the households included in the ‘comparison’ group changes our results. We focus, as described above, on those that are more similar to the capped group than the unrestricted comparison group used in the main analysis. Each model is in the same direction and the difference‐in‐differences estimates are of approximately the same size (see Appendix S8).

Third, we also explore a number of other restrictions to our data to examine whether the association is stable across various sub‐groups. In each case, we find that being at high risk of being capped increases the prevalence of mental ill health (Appendix S9). We also find that excluding those potentially affected by the two‐child limit does not alter our findings (Appendix S10).

**FIGURE 5:**
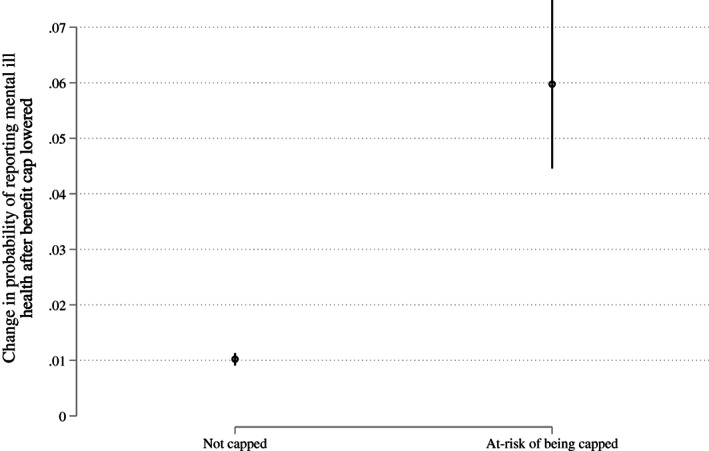
Probability of reporting mental ill health increased more among those at risk of being capped than everyone else after the benefit cap was introduced, using the Family Resources Survey to identify the at risk group. Figure based on results from Appendix S11. Data come from the Labour Force Survey and the Family Resources Survey. Vertical lines represent 95% confidence intervals

Finally, we report on the results from our FRS model of benefit cap risk, which attempts to address the problem of identifying capped families in the LFS. We use the predicted probabilities from our FRS model to analyse the association between the benefit cap and mental health in a variety of ways, described in detail in Appendix S4. Here, we report briefly the results of using a cut‐off threshold in the probability of being capped. We assume the capped individuals are those who have an estimated probability of being affected by the benefit cap greater than 0.1 (for details of how this optimal cut‐off was chosen, see Appendix S11). We conduct a difference‐in‐difference analysis similar to that presented in Figure 1; the results, shown in Figure 5, are very similar in that they indicate a statistically significant increase in the probability of mental health problems for people at risk of being capped, after the introduction of the lower cap. The identified effect is somewhat larger than in Figure 1, which would be expected if our FRS model were able to more precisely identify those at risk of being capped, and suggests that, if anything, our main estimates are conservative. We also run a series of additional models to test the consistency of our results and these are reported in Appendix S11. All the results from the FRS model are consistent with our findings with the simpler risk model from the LFS.

## 4 DISCUSSION

In November 2016, the UK government implemented a more restrictive benefit cap that cut the total amount of cash assistance that could be received by a family. We exploit the timing of this policy to examine the effect of this reduction in total income on mental health. Two key findings emerge from our analysis. First, we find that the policy increased the risk of experiencing depressive‐like symptoms among those affected by the cap. Second, we find that these negative effects on mental health emerge over a number of months. By the end of our study period, the risk of experiencing mental ill health among those at risk of being capped had increased by around 10 percentage points, a relative increase of around 50%. To put this into perspective, in November 2019 there were ~76,000 households being capped (DWP, 2020). Our estimates suggests that at least 16,000 people (~21%) in these households would have been living with depression‐like symptoms if the benefit cap had remained unchanged, with around 6600 additional people (an additional 9%) experiencing depressive‐like symptoms as a result of the lowering of the cap. This is likely to be a conservative estimate of the overall impact of the cap, for three reasons. First, it only captures the effect of the reduction in the cap and not its original introduction. Second, it excludes some of the spill‐over effects onto other family members (such as children) whose mental health is less reliably captured in the survey. Finally, our data do not allow us to identify precisely those households which are capped in practice. Sensitivity tests suggest more accurate identification would increase the estimates.

One broader implication of our results concerns the mental health effects of income shocks brought about by welfare reform. While the impact of income on physical health is still contested (Gunasekara et al., 2011), there is growing evidence using quasi‐experimental designs that increases in income can lead to reductions in depression and anxiety, especially in low‐income families (Cooper & Stewart, 2015; Cooper & Stewart, 2020). There is less evidence on the impact of *cuts* rather than increases in social security. Our study contributes vital new evidence to support the hypothesis that reductions in income can be harmful to mental health in the short‐term.

Our results are also significant for what they tell us about the nature of this particular reform. The cap most affected lone parents whose total income was close to median earnings for in‐work families. The explicit assumption underlying the policy was that this provided a reasonable and adequate ‘minimum income’ that would still allow ‘people… to take responsibility for their decisions in the light of what they can afford’ (Freud, 2013). Our results provide evidence that capped income is not in fact adequate. In practice, the families affected by the cap are very often living in relative poverty simply because of their household composition, and in addition face high housing costs. That is, while many believed that those capped received a non‐trivial sum of money from the government in absolute terms, these families were still finding it difficult to make ends meet because of high spending needs. In this respect, the concerns of some charities and religious leaders have been borne out. Pushing capped families even further into poverty has negatively affected well‐being.

If the cap is harming the mental health of parents (and particularly mothers) then this reform will have cascading effects on children's outcomes too (Wickham et al., 2017). Providing more income to low‐income families has been found to improve both cognitive, social and behavioural development in children (Cooper & Stewart, 2020). At the more extreme end, reductions in welfare payments have been linked to increased child maltreatment. Parental well‐being, and particularly maternal anxiety and depression, appear to be a key part of the mechanism linking poverty to children's outcomes (Cooper & Stewart, 2020). Thus implementing the benefit cap may deliver short‐run savings and may even induce some families into work, but there may be long‐run consequences on the life chances of the children in these families.

Our findings also have implications for the ability of the cap to successfully incentivise labour market activity, one of the policy's central goals. Observational evidence indicates that those affected by the cap were initially pursuing employment with more energy as a result of the policy, and that capped individuals did indeed move into work at a higher rate than similar individuals who were not capped (DWP, 2014). Our results do not contradict these earlier findings but instead suggest that the cap may have had heterogeneous effects. The cap may increase job search activity and even re‐employment for some of those affected, while at the same time resulting in a non‐trivial number of people experiencing poorer mental health. A formal cost–benefit analysis would be difficult using this data but we can still put these different effects into dialogue with each other. Estimates of the re‐employment effects range from 3.5 percentage points to 4.7 percentage points while the increase in mental ill health is, on average, around 2.6 percentage points but may be as high as 9 percentage points by the end of the period. It is difficult to draw strong conclusions from this comparison but the mental health effects are non‐trivial compared to the employment effects. Further, the effect on those experiencing poorer mental health may even push some people further away from the labour market (García‐Gómez et al., 2010).

These findings have implications for other countries beyond the United Kingdom. In countries like the United States, where social security has already adopted some of the principles underlying this new logic of social security, (fairly) hard limits on social security spending have already been implemented in the form of block grants to states, which limit the amount that can be spent on support for low‐income families. These limits are not targeted at specific groups, however, and have often been criticised as a result. A benefit cap like the one implemented in the UK may be a more palatable (and potentially even popular) way to achieve the same goal. Policy transfer between these contexts is not new: Britain adopted some of the principles underlying Clinton's welfare reforms, and these in turn borrowed from British debates going back to the implementation of the ‘poor laws’ in Britain in the 19th century (Somers & Block, 2005). Our results suggest other countries should be cautious about adopting the basic structure of the benefit cap. The social harms created by this system should give policymakers pause, especially in countries where the rate of mental ill health among low‐income families is already high. Moreover, our results also suggest that evaluations of welfare policy which only focus on employment effects may miss the wider impacts of these reforms and may even increase costs to other parts of the welfare state (such as health).

There are, of course, important limitations to our analysis. First, our measure of mental health is not a clinical diagnostic tool and so may contain measurement error. This is unlikely to materially affect our results, however, unless the policy change simultaneously affected how people responded to this question (Reeves et al., 2016). Second, while we have used multiple comparison groups to test the robustness of our results, the absence of a true experiment means all of our comparison groups are less than ideal (Dunning, 2012). Third, more work is needed to understand exactly how and why implementing a more restrictive cap harmed mental health, and this will likely require qualitative research that can trace the experiential aspects of the cap. Fourth, some capped households may have anticipated the benefit cap and responded to it before the policy was implemented. This is, however, unlikely to affect our results for three reasons. Our intention‐to‐treat design means that if there were anticipation effects (e.g., from anticipated income losses) then we would see them in the data and we do not. External evidence indicates capped households, whilst generally aware of the policy, were not sure what it would entail, diminishing the impact of these anticipatory effects (Finlay et al., 2013). Finally, neither of our data sets allow us to perfectly identify those who are capped. We address this limitation by adopting an intention‐to‐treat approach, which is likely more conservative in this instance, and by conducting a series of sensitivity analyses which explore different ways of identifying those at risk of being capped. These reveal that our main estimates may indeed be conservative and that the impact of the benefit cap could be even larger.

The benefit cap reminds us of the unintended consequences of policy and illustrates how they can exacerbate inequalities. The cap not only increases the risk of mental ill health but it does so among lone parents (usually women) who live in high‐rent areas. Indeed, one of the troubling aspects of the rise of this new logic of social security is how often the policies which flow from it seem to penalise lone—usually female—parents and their children (Desmond, 2012; Reeves & Loopstra, 2017). In this instance, our analysis suggests attempts to promote labour market activation through the benefit cap have had the adverse consequence of damaging the mental health of those exposed to this reform. This is significant, both in terms of understanding the impact of this specific policy, but also in contributing new evidence to support the broader hypothesis that cuts to social security benefits can be damaging to mental well‐being.

## Supporting information


**Appendix** S1: Supporting Information

## Data Availability

Data sharing is not applicable to this article as no new data were created or analyzed in this study.

## Endnote

^1^
For most families, it is receipt of housing benefit—assistance with the cost of renting a home—that pushes them into the cap. But this is not true for everyone. Couples and lone parents with around six children can also be affected by the cap even if they are not receiving housing benefit.
