# Supplementary material for: Does capping social security harm health? A natural experiment in the UK
Source: Soc Policy Adm. 2021 Sep 8;56(3):345–59. doi: 10.1111/spol.12768 (PMC9437934; doi:10.1111/spol.12768)
Supplement: Supplementary file 1 — Appendix S1: Supporting Information [file SPOL-56-345-s001.docx]

**Web Appendix**

Web Appendix 1: Descriptive data on the cumulative caseload of those subject to the benefit cap through Housing Benefit in November 2018.

Web Appendix 2: Composition of those who are at-risk of being capped before and after the reform

Web Appendix 3: Exploring the parallel trends assumption

Web Appendix 4: Method for modelling predicted probabilities of being capped in the FRS and estimating these for LFS respondents

Web Appendix 5: Impact of the benefit cap by region

Web Appendix 6: Interrupted Time Series Analysis of the impact of the benefit cap on mental health

Web Appendix 7: The introduction of the benefit cap did not increase the prevalence of other health outcomes (non-mental health)

Web Appendix 8: Exploring whether those at risk of being capped experience worse mental health than those who are exempt

Web Appendix 9: The introduction of the benefit cap and the prevalence of mental ill health among lone parents, larger households (more than 2 children), and lone parent households with more than 2 children.

Web Appendix 10: Excluding those who are potentially affected by the 2-child limit

Web Appendix 11: Estimating the effects of benefit cap using information from the LFS and the FRS

Web Appendix 1: Descriptive data on the cumulative caseload of those subject to the benefit cap through Housing Benefit in November 2018.

| **Categories** | **Number of capped housing via Housing Benefit** | **Categories** | **Number of capped housing via Housing Benefit** |
| --- | --- | --- | --- |
| Total | 196,837 | Total | 196,837 |
| *Amount of Housing Benefit Capped (£ per week)* | | *Region* |  |
| Up to £50 | 117,830 | North East | 7048 |
| £50.01 to £100 | 52,617 | North West | 16767 |
| £100.01 to £150 | 16,431 | Yorkshire and The Humber | 13708 |
| £150.01 to £200 | 5,291 | East Midlands | 10141 |
| £200.01 to £250 | 2,303 | West Midlands | 19194 |
| £250.01 to £300 | 1,192 | East | 15857 |
| £300.01 to £350 | 596 | London | 59016 |
| £350.01 to £400 | 317 | South East | 24529 |
| £400.01 and above | 265 | South West | 10793 |
|  | | Wales | 8297 |
| *Number of children* | 17,354 | Scotland | 11487 |
| 0 | 10,695 |  | |
| 1 | 29,901 | *Household type* | 17,045 |
| 2 | 68,439 | Single, no child dependant | 121,826 |
| 3 | 42,903 | Single, with child dependant(s) | 310 |
| 4 | 27,543 | Couple, no child dependant | 57,658 |
| 5 and above |  | Couple, with child dependant(s) |  |

Source: Department for Work and Pensions: Benefit Cap Statistics: Households capped to November 2018

Web Appendix 2: Composition of those who are at-risk of being capped before and after the reform

|  | Administrative data on capped households | | Among those at risk of being capped  (LFS data) | | | Compared to whole population |
| --- | --- | --- | --- | --- | --- | --- |
| Variable | End of 2016  (Before Benefit Cap made more restrictive) | End of 2018  (After Benefit Cap made more restrictive) | Before Benefit Cap made more restrictive | After Benefit Cap made more restrictive | Test of difference in means: After – Before  (p-value) | Difference-in-difference  (p-value) |
| Gender (female=1) | 93.5%^1^ | 92.3%^1^ | 94.77% | 95.46% | 0.572 | 0.549 |
| Age | 32.97^2^ | 34.22^2^ | 32.86 | 33.54 | 0.226 | 0.790 |
| Ethnicity (White British = 1) | -- | -- | 81.81% | 81.34% | 0.576 | 0.613 |
| London | 44%^3^ | 24%^3^ | 15.22% | 12.78% | 0.001 | 0.006 |
| Health problems (not depression) | -- | -- | 25.87% | 26.04% | 0.888 | 0.757 |
| Lone parent | 67%^3^ | 72%^3^ | 97.12% | 96.54% | 0.575 | 0.147 |
| Number of children | 3.4^2^ | 3.02^2^ | 2.09 | 2.13 | 0.165 | 0.015 |
| Children present | 94%^3^ | 93%^3^ | 98.99% | 99.12% | 0.593 | 0.918 |
| Single | 73%^3^ | 80%^3^ | 72.50% | 74.23% | 0.156 | 0.123 |
| Unemployed (ILO definition) | -- | -- | 23.17% | 17.21% | <0.001 | <0.001 |
| Economically inactive | -- | -- | 72.92% | 74.62% | 0.028 | 0.030 |

*Notes:* Economically inactive people are those who are not in work and who are not unemployed according to the ILO’s definition (which includes people who are not in work but who are actively seeking employment).

1 - This figures refers to single claimants only and apply to those capped through their Housing Benefit in November 2019.

2 – This figure refers to those capped through their Housing Benefit in November 2019.

3 – This figure refers to those capped through Universal Credit and Housing Benefit in November 2019.

Web Appendix 3: Exploring the parallel trends assumption

We try to test the parallel trends assumption behind the difference-in-differences models in different ways. We start by testing whether the coefficients on the treatment group in the pre-period (relative to time 0) are individually or jointly statistically significantly different from zero. We also do the same for the comparison group and the contrast between the treatment and comparison group. There are slight increases in the risk of mental ill health among the comparison group (those not at risk of being capped). These are very small (~0.5% increase) and much smaller than any observable change for the treatment group. Indeed when we compare the difference between those at risk of being capped and those not at risk, we find no change over time. Crucially, it is not just the case that the trends are parallel but, more than this, we find no clear trend at all. This confirms that the parallel trends assumption is met, a fact that we confirm with our interrupted time series analysis in Web Appendix 4.

Web Appendix 3a: Parallel trends pre-reform using OLS

|  | At-risk of being capped | Not at-risk of being capped | Difference between groups |
| --- | --- | --- | --- |
|  | (1) | (2) | (3) |
| Jan-Mar 15 | Baseline | Baseline | Baseline |
|  |  |  |  |
| Apr-Jun 15 | 0.023 (0.024) | 0.0023 (0.0015) | 0.021 (0.015) |
|  |  |  |  |
| Jul-Sept 15 | 0.016 (0.024) | 0.0035^*^ (0.0015) | 0.013 (0.015) |
|  |  |  |  |
| Oct-Dec 15 | 0.019 (0.024) | 0.0026 (0.0015) | 0.017 (0.015) |
|  |  |  |  |
| Jan-Mar 16 | -0.0065 (0.025) | 0.0053^**^ (0.0015) | -0.012 (0.016) |
|  |  |  |  |
| Apr-Jun 16 | 0.032 (0.025) | 0.0043^**^ (0.0015) | 0.028 (0.016) |
|  |  |  |  |
| Jul-Sept 16 | 0.037 (0.026) | 0.0054^**^ (0.0015) | 0.032 (0.017) |
|  |  |  |  |
| Oct-Dec 16 | 0.024 (0.027) | 0.0057^**^ (0.0015) | 0.018 (0.017) |
|  |  |  |  |
| Constant | 0.19^**^ (0.017) | 0.066^**^ (0.0010) | 0.066^**^ (0.0010) |
| Observations | 3980 | 456384 | 460364 |
| *R*^2^ | 0.0012 | 0.000053 | 0.0027 |

Standard errors in parentheses

^*^ *p* < 0.05, ^**^ *p* < 0.01

In addition, we follow recent advice from Kahn-Lang and Lang (2020) regarding how the parallel trends assumption might be tested, especially when the levels of the dependent variable vary. In this context, they suggest accounting for covariate specific trends in the model too to account for changes in the relationship between covariates and the dependent variable over time. This involves including interaction terms between covariates and our measure of time. We have estimated a series of these models which include covariate-specific trends one variable at a time. In every model we find our results are consistent with our main estimates. None of the confidence intervals cross zero whereas all of the confidence intervals cover our main point estimate.

Web Appendix 3b: Testing whether covariate-specific trends removes the difference-in-differences estimate


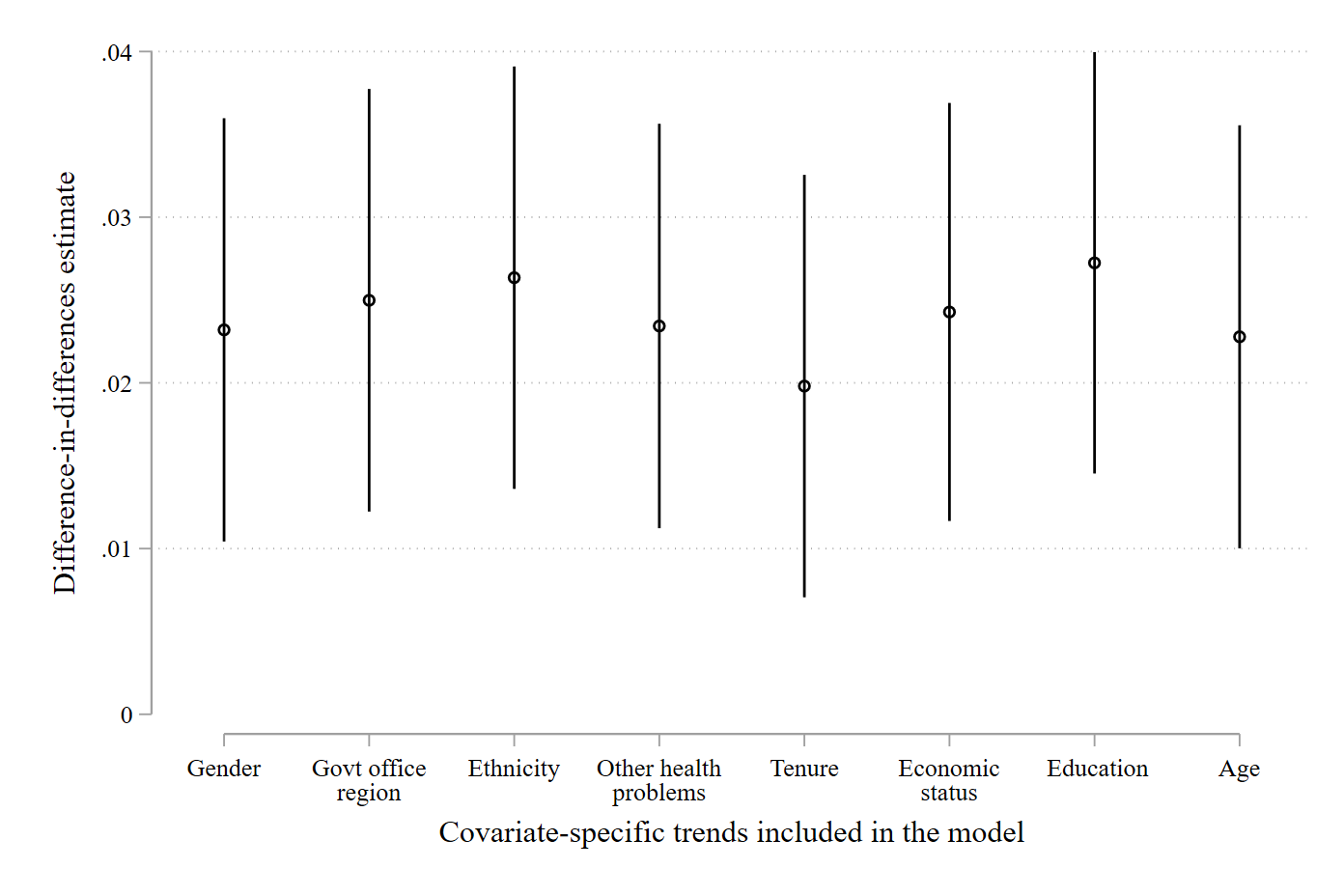


Finally, our interrupted time series analysis also includes an exploration of the parallel trends assumption (see Web Appendix 5), and this also finds that our model is consistent with that assumption.

Web Appendix 4: Method for modelling predicted probabilities of being capped in the FRS and estimating these for LFS respondents

The main limitation of the Labour Force Survey (LFS) is that it does not contain information on the income received from government cash transfers. The Family Resources Survey (FRS), by contrast, is a household survey that collects detailed data from respondents about their income from all sources. The FRS does not, however, include a measure of mental health. We therefore attempt to combine the information contained in both the LFS and the FRS to check our main results from the LFS.

For the purposes of UK government cash transfers, income is assessed at the ‘benefit unit’ level which is what we follow in this analysis. A benefit unit consists of one adult or two adults in a relationship, plus any dependent children (defined as under 16 years of age, or under 19 years of age and in full-time education) – also known in official demographic statistics as a ‘family’. The cash transfer income of a benefit unit is capped if their income from all benefits that are included in the cap exceeds the specified level.

We pool data from the 2013/14, 2014/15 and 2015/16 iterations of the cross-sectional FRS which include interviews that took place between April 2013 and March 2016 i.e. in the period after the initial benefit cap was introduced but before the more restrictive cap was implemented in November 2016. We pool data in this way to increase the number of cases identified as being affected by the cap, given the small proportion (approximately 0.24%) of UK benefit units affected^[[1]](#footnote-1)^ (ONS, 2019). For each benefit unit in the sample we sum the total income received from the cap-affected benefits, and can thus identify benefit units with benefit income that is above the November 2016 cap threshold. The FRS data also allows us to identify whether benefit units are exempt from being affected by the cap i.e. whether they are claiming any of the disability-related ‘exemption’ benefits, in sufficient hours paid employment or had worked within the past year. We are thus able to identify benefit units who would have been affected by the more restrictive cap, had it been in place before November 2016.

Using this information we develop a predictive model for whether a benefit unit is affected by the benefit cap by using variables that are both common across the LFS and FRS datasets and are related to the risk of being affected by the benefit cap. These common variables include economic status, housing tenure, number of dependent children, household composition, ethnic group, age, and government region. We develop the predictive model for benefit units who have a non-zero probability of being affected by the benefit cap (those renting their home, with dependent children, not in paid employment). We use a logistic regression for our predictive model, using 10-fold cross-validated measures of prediction error to avoid overfitting to the FRS dataset (James et al., 2013).

The final step in our predictive model is to apply this predictive logistic regression model to the LFS data for 2017 to 2018 which gives us, for each LFS case, a predicted probability of being affected by the more restrictive post-2016 benefit cap. The key assumption is that the characteristics of households who received benefits above the benefit cap level before the cap came into place is the same as those households who were actually capped when the policy came into being. This seems like a reasonable assumption given the limited behavioural change that the cap appeared to induce (Kaur et al. 2014).

*How well did this procedure work?*

The number of benefit units affected by the benefit cap, according to our calculations from the FRS, was broadly in line with that reported by national statistics. In the 2015/16 FRS sample 55 of a total of 22,540 observations were identified as having relevant cash benefit incomes above the 2016 cap threshold. Using survey weights this equates to an estimate of 64,700 benefit units affected (95% CI: 46,500-83,000), which is consistent with the 70,000 reported by official government statistics in April 2017 (see figure 1).

The results of the logistic regression model for predicting whether a benefit unit is at risk of being affected by the benefit cap are shown in Table 4.1. The results are as expected: benefit units in private rented rather than social rented housing are at greater risk of the benefit cap due to the higher rents they face, as are those living in the higher cost London and South East England regions. Benefit units with a larger number of children and those headed by someone from a minority ethnic group are also at greater risk.

The cross-validated prediction error for this model was 0.08 (i.e. 8% of cases were incorrectly classified by the model). Alternative models adding interaction terms between the prediction variables were also explored but did not significantly alter the prediction error; therefore we chose to use this relatively parsimonious prediction model.

Table 4.1: Predictive model for being capped in the Family Resources Survey

|  | Estimate | Std. Error | z value | Pr(>\|z\|) |
| --- | --- | --- | --- | --- |
| (Intercept) | -5.918 | 0.8378 | -7.064 | 1.614e-12 |
| Rented from housing association | -0.5112 | 0.2576 | -1.984 | 0.0472 |
| Rented privately unfurnished | 0.6891 | 0.2137 | 3.224 | 0.001262 |
| Rented privately furnished | 0.6812 | 0.4464 | 1.526 | 0.127 |
| Couple, two children | 0.5205 | 0.6684 | 0.7787 | 0.4361 |
| Couple, three children | 2.227 | 0.6211 | 3.586 | 0.0003361 |
| Couple, four or more children | 1.9 | 0.6614 | 2.873 | 0.004072 |
| Lone parent, one child | -0.8839 | 0.7015 | -1.26 | 0.2077 |
| Lone parent, two children | 0.6494 | 0.5961 | 1.089 | 0.276 |
| Lone parent, three children | 2.827 | 0.5769 | 4.9 | 9.584e-07 |
| Lone parent, four or more children | 3.903 | 0.5938 | 6.573 | 4.918e-11 |
| Number of children under 5 years old | 0.5012 | 0.117 | 4.285 | 1.824e-05 |
| Aged 25-34 years | 0.7073 | 0.4432 | 1.596 | 0.1105 |
| Aged 35-44 years | 0.8904 | 0.4733 | 1.881 | 0.05994 |
| Aged 45-54 years | 1.297 | 0.5422 | 2.392 | 0.01675 |
| Aged 55-64 years | 0.4396 | 1.188 | 0.37 | 0.7113 |
| North West | 0.02636 | 0.5022 | 0.05248 | 0.9581 |
| Yorks & Humber | 0.1206 | 0.5313 | 0.2269 | 0.8205 |
| East Midlands | 0.144 | 0.5322 | 0.2705 | 0.7867 |
| West Midlands | 0.1473 | 0.5105 | 0.2885 | 0.773 |
| East of England | 0.192 | 0.5504 | 0.3489 | 0.7271 |
| London | 1.393 | 0.4763 | 2.924 | 0.003454 |
| South East | 1.055 | 0.4947 | 2.134 | 0.03288 |
| South West | -0.7002 | 0.7552 | -0.9273 | 0.3538 |
| Wales | 0.3162 | 0.609 | 0.5192 | 0.6036 |
| Scotland | -0.05271 | 0.5103 | -0.1033 | 0.9177 |
| Northern Ireland | -0.001862 | 0.4756 | -0.003915 | 0.9969 |
| Mixed / Multiple ethnic groups | -0.4972 | 0.8423 | -0.5903 | 0.555 |
| Asian / Asian British | 0.7774 | 0.3711 | 2.095 | 0.03617 |
| Black African / Caribbean / British | -0.2775 | 0.3434 | -0.808 | 0.4191 |
| Other ethnic group | 1.473 | 0.4653 | 3.166 | 0.001546 |

Figure 4.2 shows the results of the predictive model for the 2,355 FRS cases with non-zero probability of being affected by the benefit cap. The predicted probabilities display a reasonable degree of differentiation between those cases that are affected by the benefit cap and those that are not. However there is a significant number of outlying cases that the predictive model identifies as having a high probability of being affected by the cap, whereas the FRS benefit claim data shows they are not in fact affected by the cap i.e. false positive predictions.

Figure 4.2: Predicted probability of being affected by benefit cap, by FRS benefit cap status


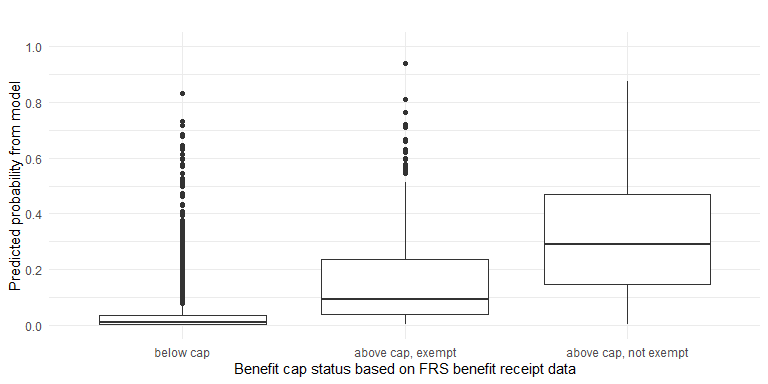


Figure 4.3 shows the predicted probabilities calculated by applying the predictive model to the 20,280 LFS observations that have non-zero probability of being affected by the benefit cap. Consistent with what we would expect, most cases have a very small probability of being affected by the benefit cap - the median probability is 0.02, the third quartile 0.09 and the maximum is 0.95. Once we transpose our model into the LFS, the model reveals that there is a small number of people with a high probability of being subject to the benefit cap.

Figure 4.3: Predicted probability of being affected by benefit cap for LFS cases


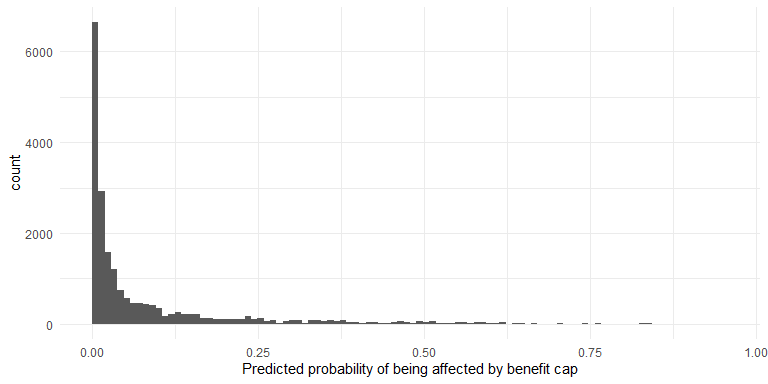


We show formal results from this approach in Web Appendix 8.

Web Appendix 5: Impact of the benefit cap by region


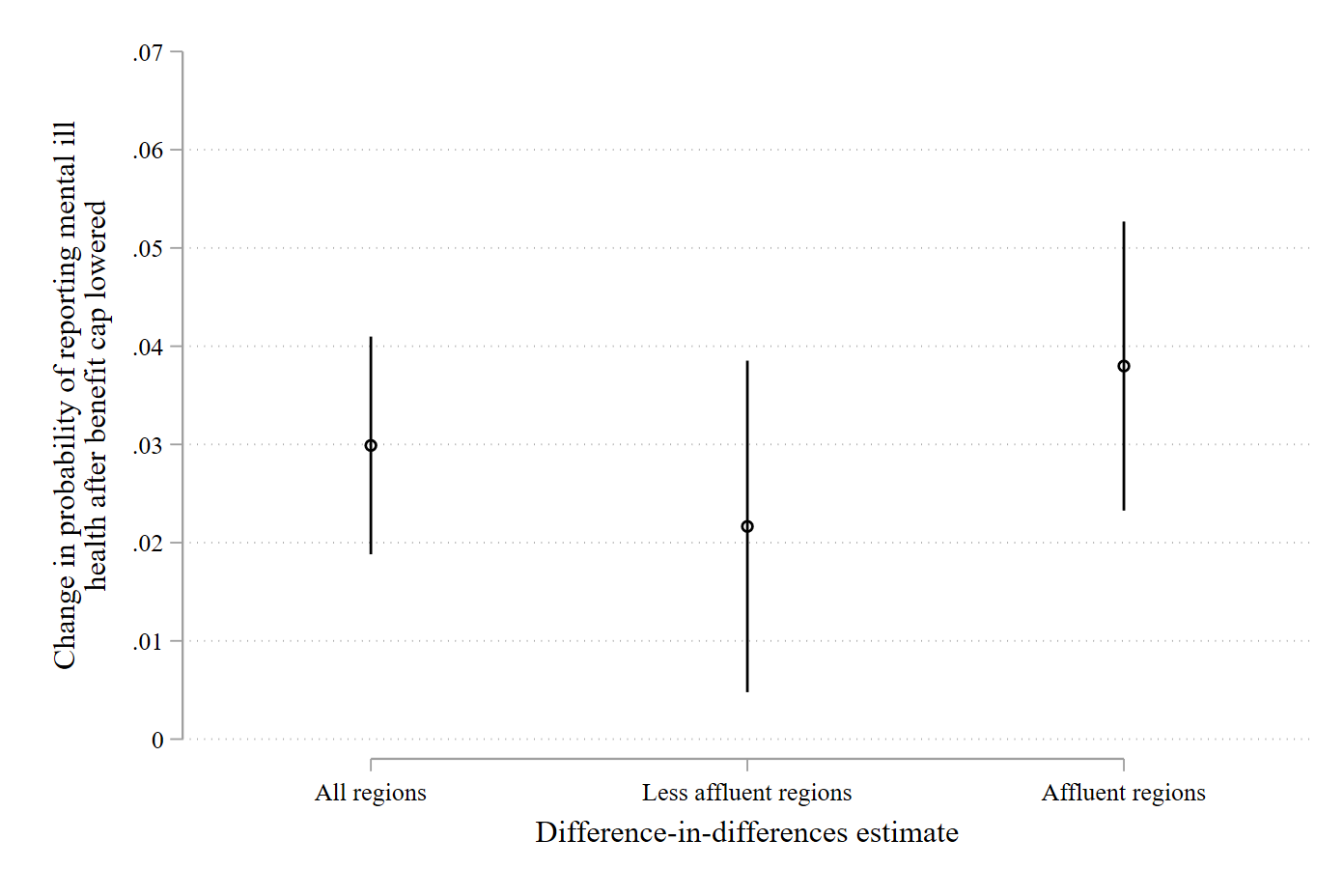


Web Appendix 6: Interrupted Time Series Analysis of the impact of the benefit cap on mental health

Below we report some of the parameters from our interrupted time series analysis, as visualised in Figure 4. We find a small positive increase in the probability of experiencing depression in the comparison group prior to intervention. But we find no difference between this slope for the treated group, suggesting that although the levels of depression are different the trends are parallel. Following the reform, there is a slight increase in the slope for the comparison group, suggesting that the probability of depression increased slowly after the benefit cap was introduced. However, we now observe a large divergence between these trends. After the benefit cap was lowered, the probability of depression increases much faster than it did before the reform for those at risk of being capped and, crucially, this slope is much steeper than the slope for the comparison group.

| Outcome: Probability of reporting depression | Point estimate | p-value | 95% CI |
| --- | --- | --- | --- |
| Pre-intervention slope for comparison group | 0.00018 | <0.001 | 0.00013 to 0.00023 |
| Difference in pre-intervention slopes between treated and comparison group | 0.00015 | 0.851 | -0.0014 to 0.0017 |
| Post-intervention slope for comparison group | 0.000088 | 0.016 | 0.000017 to 0.00016 |
| Difference in post-intervention slopes between treated and comparison group | 0.0046 | <0.001 | 0.050 to 0.051 |

*Notes:* Other parameters are estimated in the full-model but these are the essential point estimates to show the differences in the slopes before and after the trend.

Web Appendix 7: The introduction of the benefit cap did not increase the prevalence of other health outcomes (non-mental health)

|  | Probability of reporting other health problems | | |
| --- | --- | --- | --- |
|  | (1) | (2) | (3) |
| Difference-in-differences: Capped individuals after the reform | -0.0011 (0.012) | -0.0060 (0.012) | -0.0068 (0.012) |
|  |  |  |  |
| Change over time for the non-capped individuals | 0.0047^**^ (0.00095) | 0.0050^**^ (0.00091) | 0.0051^**^ (0.00091) |
|  |  |  |  |
| Difference between capped and non-capped individuals at baseline | 0.010 (0.0078) | -0.059^**^ (0.0075) | -0.059^**^ (0.0075) |
|  |  |  |  |
| Constant (probability of depression among non-capped individuals before cap lowered) | 0.25^**^ (0.00066) | -0.22^**^ (0.0080) | -0.22^**^ (0.0080) |
|  |  |  |  |
| Adjusted for covariates |  | Y | Y |
| Restrict to those who have never had mental health problem |  |  | Y |
|  |  |  |  |
| Number of individuals | 832300 | 832278 | 830584 |

*Notes:* Standard errors are reported in parentheses. Number of individuals is lower because all those with mental health issues have been excluded.

Web Appendix 8: Exploring whether those at risk of being capped experience worse mental health than those who are exempt

We focus on three exemptions.

1. We compare those at risk of being capped with those who are otherwise similar but who are not claiming social security benefits. This helps us to see what is happening to renters with similar household compositions (namely lone parents and couples with three or more children).
2. Next we compare those at risk of being capped with those who own their home or who are currently buying it through a mortgage. This helps us see what is happening to people with a similar household structure and who are claiming benefits but who are not receiving housing benefit (the main driver of the benefit cap).
3. Finally, we compare those at risk of being capped with those who have a disability. This contrast again uses this exemption to trace whether those who are capped experience a different trend in their well-being after the reform.

In all cases, we observe positive difference-in-differences estimates, suggesting that those at risk of being capped are more likely to experience worse mental health after the more restrictive cap was implemented compared to those who were not exposed or who were exempt.

1. At risk of being capped compared to those claiming no benefits.

1. At risk of being capped compared to those with a mortgage or who own their homes

1. At risk of being capped compared to those who are disabled.

Web Appendix 9: The introduction of the benefit cap and the prevalence of mental ill health among lone parents, larger households (more than 2 children), and lone parent households with more than 2 children.

|  | Probability of reporting mental health problems | | |
| --- | --- | --- | --- |
|  | Lone parents | Households with more than 2 children | Lone parent households with more than 2 children |
|  | (1) | (2) | (3) |
| Difference-in-differences: Capped individuals compared to uncapped individuals after the reform | 0.018* (0.0079) | 0.048** (0.011) | 0.035* (0.016) |
|  |  |  |  |
| Change over time for the non-capped individuals | 0.014** (0.0020) | 0.012** (0.0018) | 0.026** (0.0072) |
|  |  |  |  |
| Difference between capped and non-capped individuals at baseline | -0.0033 (0.0053) | 0.028** (0.0070) | -0.026* (0.012) |
|  |  |  |  |
| Constant (probability of depression among non-capped individuals before cap lowered) | -0.12** (0.0062) | -0.085** (0.0066) | -0.15** (0.024) |
|  |  |  |  |
| Controls for covariates | Y | Y | Y |
|  |  |  |  |
| Number of individuals | 104056 | 68867 | 9615 |

*Notes:* Standard errors are reported in parentheses.

Web Appendix 10: Excluding those who are potentially affected by the 2-child limit

Some of the families affected by the benefit cap could also be affected by the 2-child limit, a policy which limits the receipt of child tax credits and the child element of Universal Credit to the first two children (albeit with some exemptions). The 2-child limit also reduces incomes and so could affect mental health. To address this possibility, we removed families from the analysis that may have been subject to the 2 child limit. We do this by: 1) excluding those who had at least 1 child under the age of 1 and that had 3 or more kids and were interviewed 3 months or more after the 2 child limit was introduced, 2) excluding those who had at least 1 child under the age of 1 and that had 3 or more kids (irrespective of when they were interviewed) and 2) excluding those who had at least 1 child under the age of 2 and that had 3 or more kids (again irrespective of when they were interviewed). This diverse approach gives us a sense of how more and less conservative approaches to this issue affect our analysis. The results are reported in the figure below.


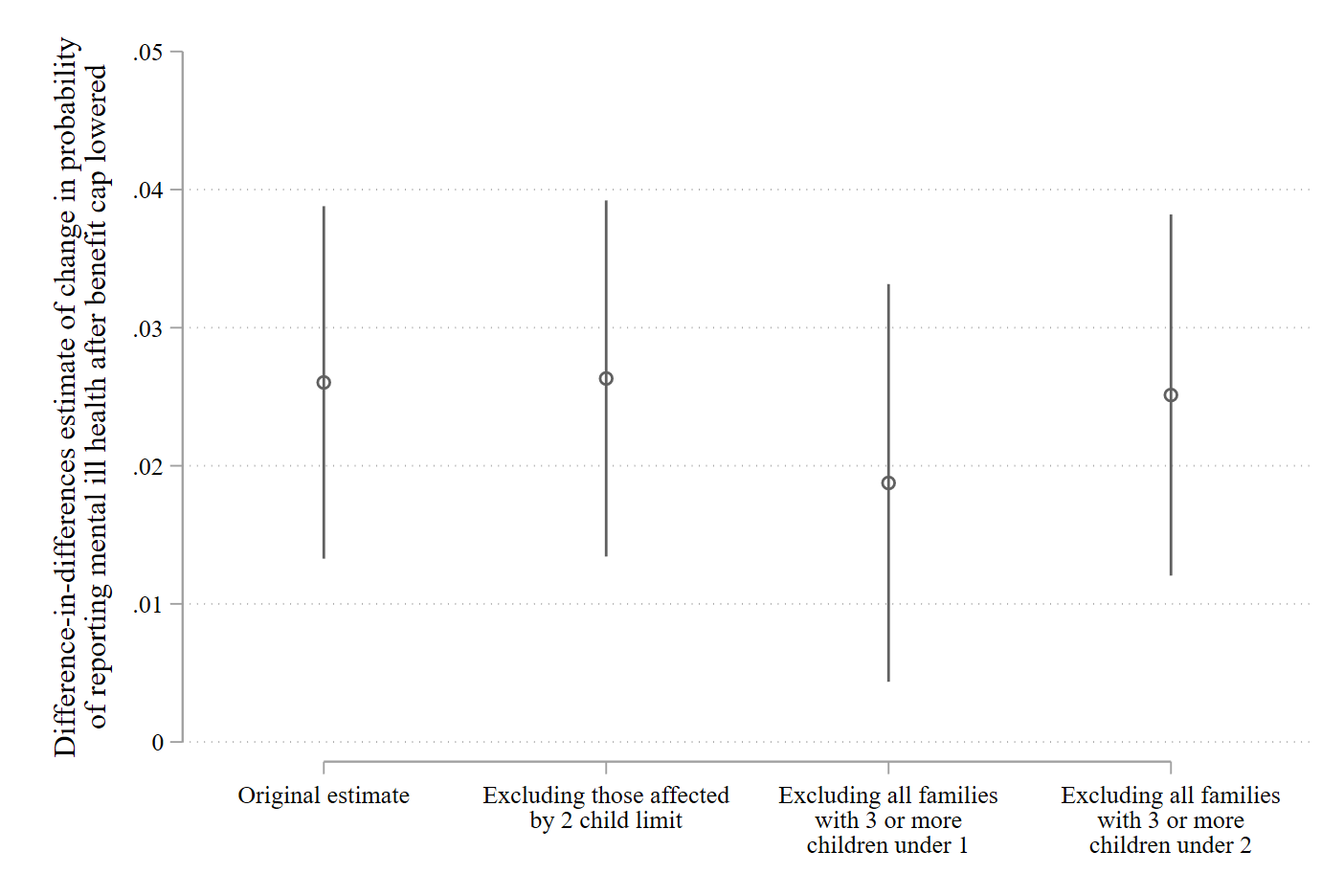


The first estimate replicates the difference-in-difference estimate reported in figure 2 and table 1 in the main text. The next three estimates of the difference-in-difference impose certain restricts by excluding particular households with young children. We find that our estimates are stable across these exclusions, even in the final and most restrictive model.

Web Appendix 11: Estimating the effects of benefit cap using information from the LFS and the FRS

In Web Appendix 4, we described the methods we used to derive the predicted probability of being capped for each individual in the LFS using information available to us in the FRS. Once we have the predicted probability of being subject to the benefit cap, we can then use different approaches to identifying the effect of the benefit cap.

*Binary classifier approach*

Our first approach is to convert the predicted probability into a 0/1 classifier to identify LFS cases at high risk of being affected by the benefit cap. To identify the optimal classifier we create a series of 100 binary classifiers by using probability cutoffs ranging from 0.01 to 1 in increments of 0.01. In the first iteration of these classifiers (cutoff 0.01), those benefit units with predicted probability greater than 0.01 would be classified as being at risk of being affected by the benefit cap. In the FRS data we compare this classification to the ‘true’ classification derived from the FRS benefit income calculations, thus determining whether each classification is a true positive or false positive. We choose an optimal cutoff probability by plotting a ‘ROC curve’ of true positive rate ($tpr$) vs false positive rate ($fpr$), and identifying the point at which $tpr=(1-fpr)$. The true positive rate is the proportion of benefit cap cases that are correctly identified as such; the false positive rate is the proportion of non benefit cap cases that are incorrectly identified as benefit cap cases.

We use this optimal cutoff probability to assign each case in the LFS data to a binary classification. This allows us to, in a similar fashion to our simple LFS classifier, calculate a simple difference-in-difference estimate of the policy impact and plot the prevalence of reported mental health problems over time for the at risk group compared to the not-at-risk group. In addition we model the evolution of mental health problems over time using a linear regression model of the form

$$\text{Health}_{i,t}=\alpha+\beta_{1}\text{Capped}_{i,t}+\beta_{2}\text{time}+\beta_{3}\text{Capped}_{i,t}\text{time}+$$

$$\beta_{4}\text{Policy}_{t}\text{Uncapped}_{i,t}\text{time}+\beta_{5}\text{Policy}_{t}\text{Capped}_{i,t}\text{time}$$

In this model the coefficient of interest is $\beta_{5}$, the average additional quarterly prevalence increase after the reform was introduced, for capped individuals.

Figure 10.1 shows the ROC curve that plots the true positive rate $tpr$ against the false positive rate $fpr$ for 101 values of the cutoff probability for classifying a case as being affected by the benefit cap, varying from a cutoff probability of 0 to 1 in increments of 0.01. The optimal classifier where $tpr=(1-fpr)$ can be read from the results table as a cutoff probability of 0.1, at which point $tpr$ is 0.84 and $fpr$ is 0.16. This is a binary classifier in which all LFS cases with predicted probability of being affected by the benefit cap greater than 0.1 are classified as benefit cap cases, those equal to or below the cutoff are classified as non benefit cap cases.

Figure 10.1: ROC curve for binary classifiers


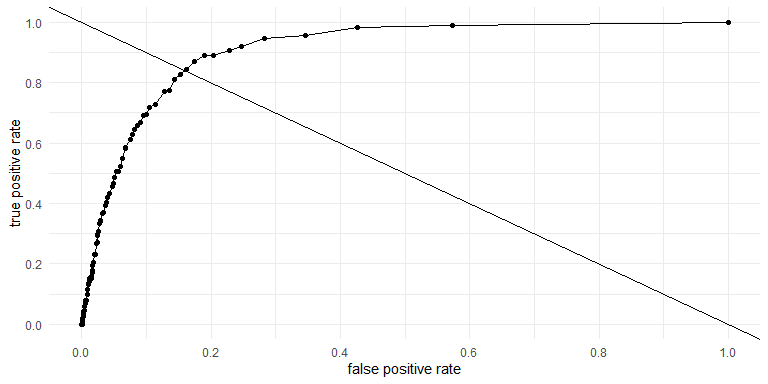


In an analogous fashion to the model in Figure 4 (in the main paper) we estimate an unadjusted difference in difference model for the change in mental illness prevalence after the benefit cap was introduced for people at risk of being capped compared to those not at risk of being capped. The results shown in Figure 4 (in the main paper) display a similar pattern to those for our simpler benefit cap risk indicator, although the effect size is larger than the estimate from our original model (β = 0.060, (95% CI: 0.045-0.075). If anything, then, our main estimates are likely to be conservative.

Figure 10.2 below replicates the analysis of figure 2 earlier in our paper. Again we find that the level of mental ill health before the reform was relatively stable amongst those at risk of being capped, with a steady rise following the lowering of the benefit cap.

Figure 10.2: Introduction of the benefit cap and the prevalence of mental ill health among those who are at-risk of being capped and those who are not, by quarter - FRS-based binary indicator


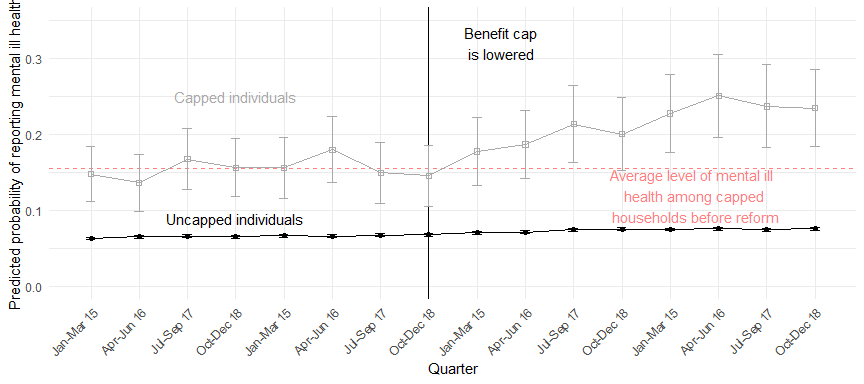


Table 10.3 shows the results of the linear regression model that fits the prevalence of self-reported mental ill health as a function of time, with separate slopes fitted for the capped and uncapped groups, before and after the reform. The only slope in the model that is significantly different from zero is the additional quarterly prevalence increase after the reform for capped individuals (last row in the table).

Table 10.3: Linear regression of mental ill health time series

|  | Estimate | Std. Error | t value | Pr(>\|t\|) |
| --- | --- | --- | --- | --- |
| Prevalence among uncapped individuals in first quarter | 0.06455 | 0.006268 | 10.3 | 1.146e-10 |
| Difference between capped and uncapped individuals in first quarter | 0.08201 | 0.008864 | 9.251 | 1.045e-09 |
| Average quarterly prevalence increase for uncapped individuals, period before reform | 0.0004924 | 0.001527 | 0.3225 | 0.7496 |
| Average additional quarterly prevalence increase for capped individuals, period before reform | 0.001524 | 0.002159 | 0.7058 | 0.4866 |
| Average additional quarterly prevalence increase after reform, uncapped individuals | 0.0003366 | 0.001178 | 0.2857 | 0.7774 |
| Average additional quarterly prevalence increase after reform, capped individuals | 0.004171 | 0.001178 | 3.54 | 0.001531 |

*Examining the effect of the benefit cap using all the information from the predicted probabilities*

A drawback of the ‘binary classifer’ approach is that it discards information about the probability of a case being affected by the benefit cap. For example, with a binary classifer cutoff probability of 0.1, a case with predicted probability 0.2 is treated the same as a case with predicted probability 0.5. Our second approach is to use the full information from the predicted probabilities by using them as a measure of treatment intensity. We estimate a model of the form

$$\text{Health}_{i,t}=\alpha+\gamma_{1}\text{AtRiskofCapping}_{i,t}+\gamma_{2}\text{time}+\gamma_{3}\text{CappingProbability}_{i,t}+$$

$$\gamma_{4}\text{CappingProbability}_{i,t}\text{time}+\gamma_{5}\text{Policy}_{t}\text{CappingProbability}_{i,t}\text{time}$$

In this model the coefficient of interest is $\gamma_{5}$, the additional marginal quarterly increase in prevalence of mental ill health for a unit increase in the probability of being affected by the benefit cap, after the reform was introduced. The coefficient $\gamma_{4}$ is used to test whether there was any such relationship before the reform was introduced.

Table 10.4 shows the results from the linear regression of mental ill health prevalence on time and the probability of being affected by the benefit cap. The key result is in the final row of the table, which shows a positive and statistically significant relationship between the probability of being affected by the benefit cap and mental ill health prevalence, in the period after the benefit cap reform was introduced. By contrast, there is no statistically significant relationship between capping probability and increase in mental ill health in the period before the reform was introduced.

Table 10.4: Linear regression of increase of mental ill health prevalence on probability of being affected by the benefit cap

|  | Estimate | Std. Error | t value | Pr(>\|t\|) |
| --- | --- | --- | --- | --- |
| Prevalence among uncapped individuals in first quarter | 0.06688 | 0.0002436 | 274.6 | 0 |
| Average quarterly increase in mental ill health prevalence for all individuals | 0.0009734 | 5.203e-05 | 18.71 | 4.246e-78 |
| Difference in first quarter between uncapped individuals and those with non-zero probability of being capped | 0.2084 | 0.002098 | 99.32 | 0 |
| Marginal increase in prevalence of mental ill health from increase of 0.1 in probability of being affected by the benefit cap | -0.03867 | 0.002322 | -16.65 | 2.843e-62 |
| Marginal quarterly increase in prevalence of mental ill health from increase of 0.1 in probability of being affected by the benefit cap - before reform was introduced | -0.0009691 | 0.0005508 | -1.759 | 0.07849 |
| Additional marginal quarterly increase in prevalence of mental ill health from increase of 0.1 in probability of being affected by the benefit cap - after reform was introduced | 0.003756 | 0.000953 | 3.941 | 8.122e-05 |

Figure 10.5 displays the predicted evolution of mental ill health prevalence over time in the period before and after the benefit cap reform was introduced, according to the fitted model in Table 10.4. It shows the evolution of mental ill health for individuals with zero probability of being capped against those with a probability of 0.28 of being capped. This is chosen for comparison with the binary indicator approach above, as the mean predicted probability of being capped for those individuals above the binary cutoff of 0.1. As can be seen, the model predicts an increase in mental ill-health prevalence for those at risk of being subject to the benefit cap, in a similar relationship to that shown in the binary indicator model of Figure 10.2.

Figure 10.5: Introduction of the benefit cap and the prevalence of mental ill health among those who are at-risk of being capped and those who are not, by quarter - FRS-based binary indicator


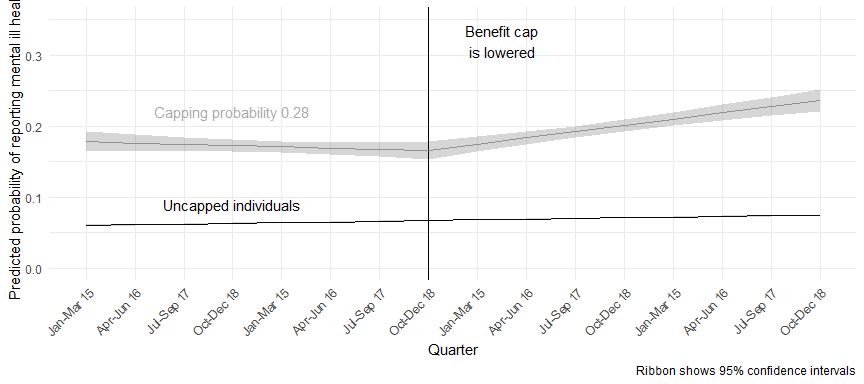


*Exploring differences between those at risk of being capped in the FRS and those at risk of being capped in the LFS*

These two different approaches to specifying who is and who is not at risk of being capped identify different groups of people. The LFS-based approach identifies 6824 households at risk and the FRS-based approach identifies around 4684. There is some overlap between these two approaches, that is, around 1498 people are found in both specifications. But this means that each measure is identifying some individuals that are not captured by the other. Here we explore these groups and then consider whether our estimates are different depending on the combination we pay most attention to.

First, we explore descriptive differences. As noted above, our LFS-based measure has some similarities to administrative data in terms of gender, age, and the presence of children in the household. But it was less accurate in terms of the number of people based in London, the number of children in the household and whether households were lone parents. When we compare the LFS-based measure and the FRS-based measure, we find that the FRS-based measure corrects for some but not all of these biases. The FRS-based measure is more accurate when it comes to the proportion of capped households based in London and the number of children in the household. But the FRS-based measure was less accurate in terms of the number of women, the number of single households, and the number of lone parents, compared to the administrative data. Neither is perfect, therefore, but both have strengths.

Next, we explore the difference-in-differences estimates using these two measures together. We use those who are categorized as not at risk of being capped in both measures as our baseline (or comparison group). We then compare this group to: 1) those only identified as being at risk in the LFS-based measure (the main one used in the paper), 2) those only identified as being at risk in the FRS-based measure (the sensitivity analysis), and 3) those identified as being at risk in both measures.

What these results show us is that the LFS-based sample potentially has a smaller difference-in-differences estimate than the FRS-based estimates, which are both slightly higher, albeit that the indicator using both FRS and LFS to identify individuals has wide confidence intervals which overlap with the LFS-only measure (*p* = 0.19). In short, whichever way we cut the data, we see a clear pattern: those at risk of being capped (defined in various ways) consistently see their mental health worsen over this period.

Figure 10.5: Exploring the difference between FRS-based and the LFS-based measures

Viewed together, these results reinforce the primary message from our main results. We find a consistent and pronounced increase in the risk of mental ill health after the introduction of the benefit cap, among those who are risk of being affected by it.

1. Authors’ calculation from published official statistics. There were 29.0 million benefit units in Great Britain in mid-2017 (ONS, 2019) of which 68,900 were subject to the benefit cap, or 0.24%. [↑](#footnote-ref-1)
